# Disclosure of salicylic acid and jasmonic acid-responsive genes provides a molecular tool for deciphering stress responses in soybean

**DOI:** 10.1038/s41598-021-00209-6

**Published:** 2021-10-18

**Authors:** Sebastian F. Beyer, Paloma Sánchez Bel, Victor Flors, Holger Schultheiss, Uwe Conrath, Caspar J. G. Langenbach

**Affiliations:** 1 Plant Biochemistry & Molecular Biology Unit, Department of Plant Physiology, RWTH Aachen University, 52074 Aachen, Germany; 2 Metabolic Integration and Cell Signaling Laboratory, Plant Physiology Department of CAMN, Universitat Jaume I, 12071 Castellón, Spain; 3 Agricultural Center, BASF Plant Science Company GmbH, 67117 Limburgerhof, Germany

**Keywords:** Plant hormones, Plant signalling, Plant stress responses

## Abstract

Hormones orchestrate the physiology of organisms. Measuring the activity of defense hormone-responsive genes can help understanding immune signaling and facilitate breeding for plant health. However, different from model species like Arabidopsis, genes that respond to defense hormones salicylic acid (SA) and jasmonic acid (JA) have not been disclosed in the soybean crop. We performed global transcriptome analyses to fill this knowledge gap. Upon exogenous application, endogenous levels of SA and JA increased in leaves. SA predominantly activated genes linked to systemic acquired resistance and defense signaling whereas JA mainly activated wound response-associated genes. In general, SA-responsive genes were activated earlier than those responding to JA. Consistent with the paradigm of biotrophic pathogens predominantly activating SA responses, free SA and here identified most robust SA marker genes *GmNIMIN1*, *GmNIMIN1.2* and *GmWRK40* were induced upon inoculation with *Phakopsora pachyrhizi,* whereas JA marker genes did not respond to infection with the biotrophic fungus. *Spodoptera exigua* larvae caused a strong accumulation of JA-Ile and JA-specific mRNA transcripts of *GmBPI1*, *GmKTI1* and *GmAAT* whereas neither free SA nor SA-marker gene transcripts accumulated upon insect feeding. Our study provides molecular tools for monitoring the dynamic accumulation of SA and JA, e.g. in a given stress condition.

## Introduction

To survive hostile conditions, sessile plants depend on the flawless perception and fine-tuned response to signals in the environment^1,2^. For example, plants have evolved a highly flexible immune system of receptors of microbial-patterns and effectors as well as interactive defense mechanisms to perceive and respond to microbial pathogens. *Arabidopsis thaliana* (subsequently referred to as Arabidopsis), for example, mostly fends off biotrophic and hemibiotrophic pathogens by salicylic acid (SA)-induced defense responses whereas it fights necrotrophic pathogens and herbivorous insects mainly by defense reactions that are activated by jasmonic acid (JA) and its derivatives [collectively referred to as jasmonates (JAs) and ethylene (ET)]^3,4^. Synergistic and antagonistic crosstalk of the SA and JA/ET signaling pathways adds additional complexity and flexibility to the plant immune system^3–5^. In their crosstalk, SA mostly overrules JA^6–9^, for example by inhibition of transcription factors involved in JA signaling and/or by inducing transcription-repressive epigenetic marks to histones (e.g. trimethylation of lysine 27 in histone H3) in JA-responsive defense genes (e.g. *PDF1.2*)^10^. Only few studies reported the suppression of SA signaling by JA^11,12^ or equal importance of the two hormone pathways^13^. While SA and JA/ET signaling are the best studied plant defense pathways^3^, those of abscisic acid^14^, β-aminobutyric acid^15^ auxins^16^, brassinosteroids^17^, cytokinins^18^, gibberellins^19^, nitric oxide^20^, and N-hydroxy-pipecolic acid^21^ are also involved in plant defense signaling.

In Arabidopsis, stimulation of the SA pathway causes induction of multiple genes with presumed function in local and systemic immune responses (e.g. *PR1, PR2, PR5, EDS1, PAD4, SAG101, ALD1, WRKY 70, SARD1*)^4,22–24^. Different from SA, JA and the natural ET precursor 1-aminocyclopropane-1-carboxylate (ACC) do not activate *PR1* in Arabidopsis^25^. They rather trigger two distinct, but interconnected pathways that synergistically activate *PLANT DEFENSIN* (*PDF*) *1.2* expression^26,27^. However, only the JA signaling pathway also stimulates *VEGETATIVE STORAGE PROTEIN* (*VSP*) *1* gene activity^27^ and *VSP1* activation by JA is attenuated in the presence of ET^28^. Thus, recording the activity or inactivity of marker genes in specific hormone signaling pathways in a given plant may enable conclusions as to the spectrum of diseases against which a certain treatment may cause resistance and, thus, secure yield. Regrettably, most studies about the molecular activation and interaction of phytohormone pathways were done in model species such as Arabidopsis rather than in crops such as soybean. SA and JAs are both present in the soybean crop^29,30^ but, despite soybean’s importance as a food and feed source^31^, little is known about the SA/JA signaling pathways and the identity of SA and JA target genes in this plant. Knowledge of their identity may help providing stress-resilient soybean cultivars.

Here, we performed a comparative, and global transcriptome analysis and we identified robust marker genes for SA and JA signaling in the soybean crop. We validated usability of the genes for use as diagnostic tools to determine SA or JA responsiveness in soybean upon exposure to two distinct biotic stresses, that is inoculation with *Phakopsora pachyrhizi* (*Pp*), which causes Asian soybean rust^32,33^ and leaf feeding of *Spodoptera exigua* Hübner^34^.

## Results

### Recording SA and the SA-specific leaf transcriptome

**Figure 1:**
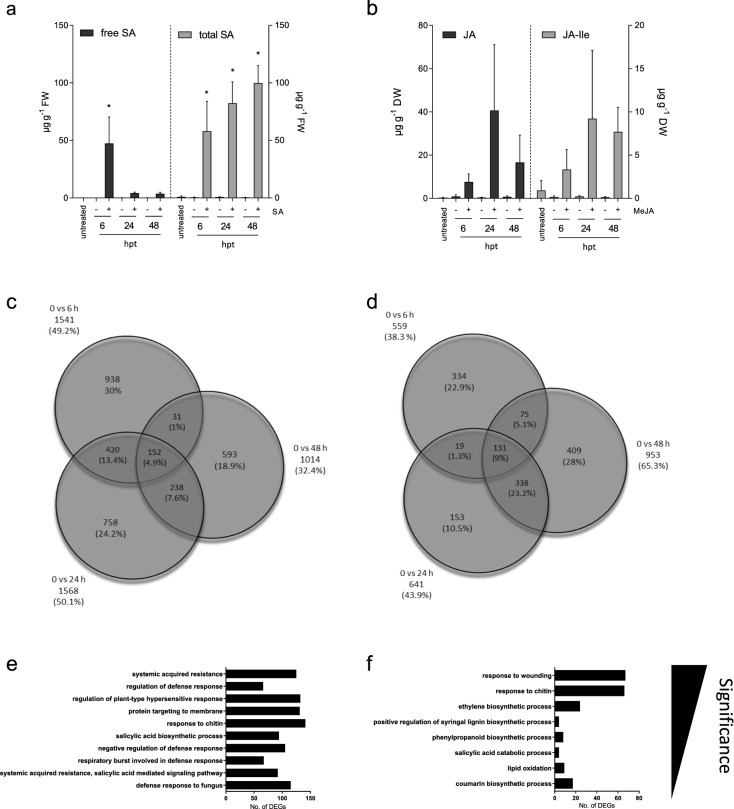
Verification of phytohormone uptake and temporal activation of gene expression. (**a**) Accumulation of free (black bars) and total (grey bars) SA in soybean leaves upon soil drench with water (−) or a 1 mM solution of SA (+) as quantified by HPLC analysis. Average values and error bars of three independent experiments with four individual plants per treatment and experiment are shown. Asterisks indicate significant differences to the adequate untreated control (Dunnett's multiple comparisons test; p < 0.0001). (**b**) JA (black bars) and bioactive JA-Ile (grey bars) in soybean leaves in the absence (−) or presence (+) of MeJA as determined by LC–MS analysis. Average values and error bars of three independent experiments with three individual plants per treatment and experiment are shown. Significance was calculated by using Dunnett's multiple comparisons test. No significance differences occurred. (**c**, **d**) Venn diagrams displaying the number of genes with significantly (p ≤ 0.01) induced expression after (**c**) SA treatment and (**d**) exposure to MeJA compared to the untreated control based on RNA-seq data. In addition to the absolute numbers, the relative amount of induced genes is shown in brackets. Identical samples were used for phytohormone quantification and RNA-seq. (**e**, **f**) GO terms overrepresented among (**e**) SA-induced (**f**) or MeJA activated genes sorted according to their corrected p-value (Bonferroni correction) (p < 0.05).

To identify reliable SA marker genes in soybean we watered soil-grown plants at V2 stage with 150 ml of an aqueous SA solution (1 mM). We verified the uptake of SA by quantifying the level of free and total SA in the first trifoliate leaf by HPLC analysis. Figure 1a shows that the SA drench led to a ~ 400-fold increase in free SA within 6 h with a maximum of ~ 50 µg g^−1^ free SA. At 6 h after treatment (hpt), the level of total SA was only slightly higher than that of free SA, suggesting that SA was mainly present in its free form (82%). The level of free SA was much lower at 24 and 48 hpt than at 6 hpt, but still 30–40-fold higher than in the water-treated controls (Fig. 1a). The decrease in free SA at later times coincided with a further increase in total SA up to ~ 100 µg g^−1^ FW at 48 hpt (Fig. 1a). At 24 and 48 hpt bound SA (calculated as the difference between total and free SA) contributed the biggest part to total SA (~ 5% free SA and ~ 95% bound SA) (Fig. 1a).

To gain comprehensive knowledge of the changes in gene expression following SA accumulation, we performed global transcriptome analyses. For read statistics of the RNA-seq analysis, please refer to Tables S1 and S2. The analysis identified 3130 genes with induced expression upon SA treatment (Fig. 1c). Because we aimed to identify genes that may serve as a molecular marker to hormone treatment we here focused on genes that were activated upon accumulation of free SA in leaves but disregarded genes whose expression was suppressed by SA. Their identity is given in Fig. S1. In close correlation with the high levels of free SA at 6 hpt and its low levels at 48 hpt (Fig. 1a), the overall response to SA was highest at 6 and 24 hpt with 1541 and 1568 significantly activated genes and lower at 48 hpt with 1014 significantly activated genes (Fig. 1c). While free SA levels severely dropped between 6 and 24 hpt (Fig. 1a), the number of SA-activated genes remained high during this period (Fig. 1c).

### Recording JA and the JA-specific transcriptome in leaves

To identify JAs-responsive marker genes in soybean, we exposed plants to MeJA.^.^ Subsequently we measured the accumulation of JA and its bioactive derivative JA-Ile^35,36^ in leaves at various times after exposure. As seen in Fig. 1b, the accumulation in leaves of JA and JA-Ile had an apparent maximum at 24 h after exposure to MeJA constituting ~ 40 µg g^−1^ dry weight (DW) for JA and 9 µg g^−1^ DW for JA-Ile (Fig. 1b). After 24 h, the MeJA-enhanced JA and JA-Ile levels dropped. However, the observed decrease in JA-Ile between 24 and 48 hpt was less pronounced than that observed for free JA (Fig. 1b). The accumulation of mRNA transcript of *GmVSP-B* (*Glyma.08g200100*), which contains a MeJA-responsive domain in its promoter region^37^, also pointed to similar kinetics of JA presence in MeJA-exposed plants. In fact, *GmVSP-B* expression was activated at all time points following MeJA exposure (Fig. S2).

Global transcriptome analysis of MeJA-exposed plants identified 1459 genes whose expression was significantly activated by MeJA (Fig. 1d). Gene activation by MeJA followed JA-Ile accumulation (Fig. 1b,d). At 6 hpt, 559 genes were significantly more expressed in MeJA-treated plants than in the controls. The number of activated genes increased with time constituting 953 genes at the 48 hpt time point. The identity of genes whose expression was suppressed by MeJA treatments is given in Fig. S1.

### Gene ontology (GO)-enrichment analysis

GO-enrichment analysis revealed that among the SA-induced genes those associated with systemic acquired resistance (SAR) were the most significantly enriched loci (Fig. 1e). However, various genes in some other SA signaling-related GO terms were enriched as well. They include “regulation of defense responses”, “regulation of plant hypersensitive response”, “SA-biosynthetic process”, “SA-mediated signaling pathway” and “defense response to fungus” (Fig. 1e).

GO categories that were overrepresented among the MeJA-induced genes comprised “response to wounding” and “response to chitin” (Fig. 1f), the latter also being enriched upon SA treatment (Fig. 1e). Among genes in other GO terms, MeJA activated the expression of genes in the “ethylene biosynthetic process”, “phenylpropanoid biosynthetic process”, “SA catabolic process” and “lipid oxidation” bins (Fig. 1f). In addition to the genes whose expression was specifically activated by either SA or MeJA, multiple genes in the GO categories associated with primary metabolism (e.g. photosynthesis) were repressed by any of the two hormones (Fig. S1). The overall pattern of gene activation upon treatments might reflect the reported growth-to-defense transition upon exposure to SA or JAs^38–40^.

### Identification of genes that are specifically activated by SA or JA

**Figure 2:**
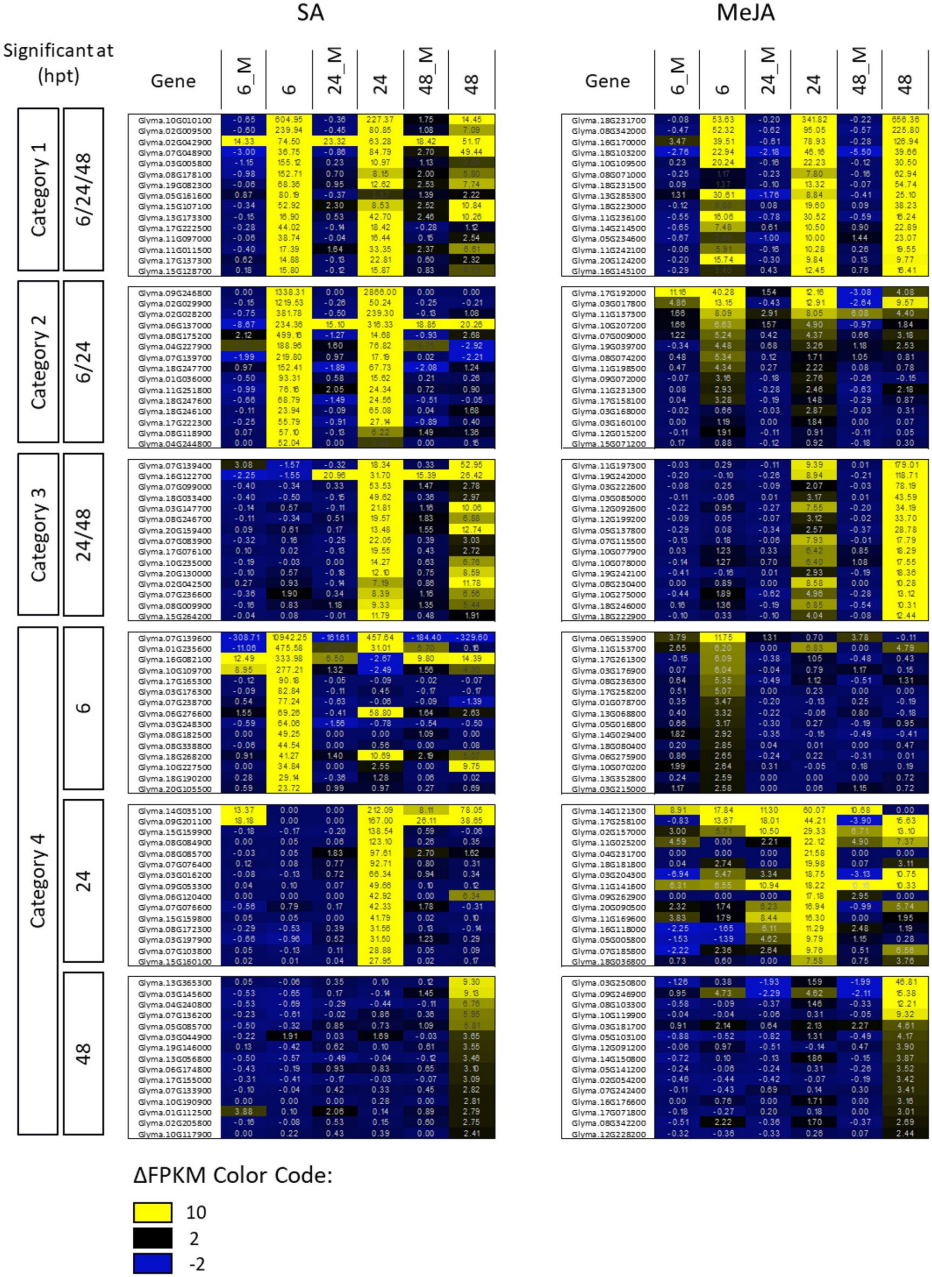
SA or JAs-responsive soybean genes classified by their temporal induction pattern. Leaf transcriptomes were recorded by RNA-seq in SA, MeJA, mock (M)-treated or untreated soybean leaves at the indicated time points. Specified are genes with significantly (p ≤ 0.01) induced expression after treatment with either SA or MeJA for more than one (category 1–3) or at a single time point of sampling (category 4) compared to untreated controls. In addition, only those genes were included that were not significantly differently expressed among untreated and mock-treated samples. Numbers correspond to ΔFPKM values that were highlighted according to the displayed color code.

To identify genes whose expression is specifically activated by either SA or JAs we compared the gene expression profiles in trifoliate leaves of soybean plants at 6, 24 and 48 hpt with SA or MeJA and compared it to that of untreated control leaves. We then selected genes that were significantly induced (p ≤ 0.01) by only one of the two hormones. From these sets of genes, we discarded those genes that also were differentially expressed among mock-treated and untreated plants, independent of the time point of differential expression. To identify marker genes that, in addition to strong induction, would exhibit high expression upon treatment, we sorted identified genes by the difference in fragment per kilobase per million mapped reads (FPKM)-values between the untreated control and treatments (ΔFPKM). Figure 2 depicts genes whose transcript levels are stable in all control treatments but significantly increased to high levels only with exclusively one of the two hormones applied. These candidate genes were further divided into categories of top 15 genes with induced expression at all tested time points after treatment (category 1), early responsive genes with induced expression after 6 and 24 hpt (category 2), late responsive genes with increased expression after 24 and 48 hpt (category 3), and genes that were induced only at one single time point after treatment (6 or 24 or 48 hpt) (category 4) (Fig. 2). Consistent with the number of genes induced by SA or MeJA (Fig. 1c,d), the induced expression of genes by SA was more pronounced at the early than late time points. The opposite was true for the genes induced by MeJA that had their peak in expression at later time points (Fig. 2). For example, SA-activated genes were induced stronger in category 2 compared to category 3, whereas mRNA transcript abundance of MeJA-activated genes was mostly lower in category 2 than in category 3 (Fig. 2). Taken together, the results in Fig. 2 revealed that exogenously applied SA and MeJA elicit distinct temporal gene expression patterns in soybean, with SA-responsive genes being activated preferably at the early and MeJA-induced genes at the later times upon treatment.

### Verifying specific responsiveness of identified marker gene candidates to SA or JAs

**Figure 3:**
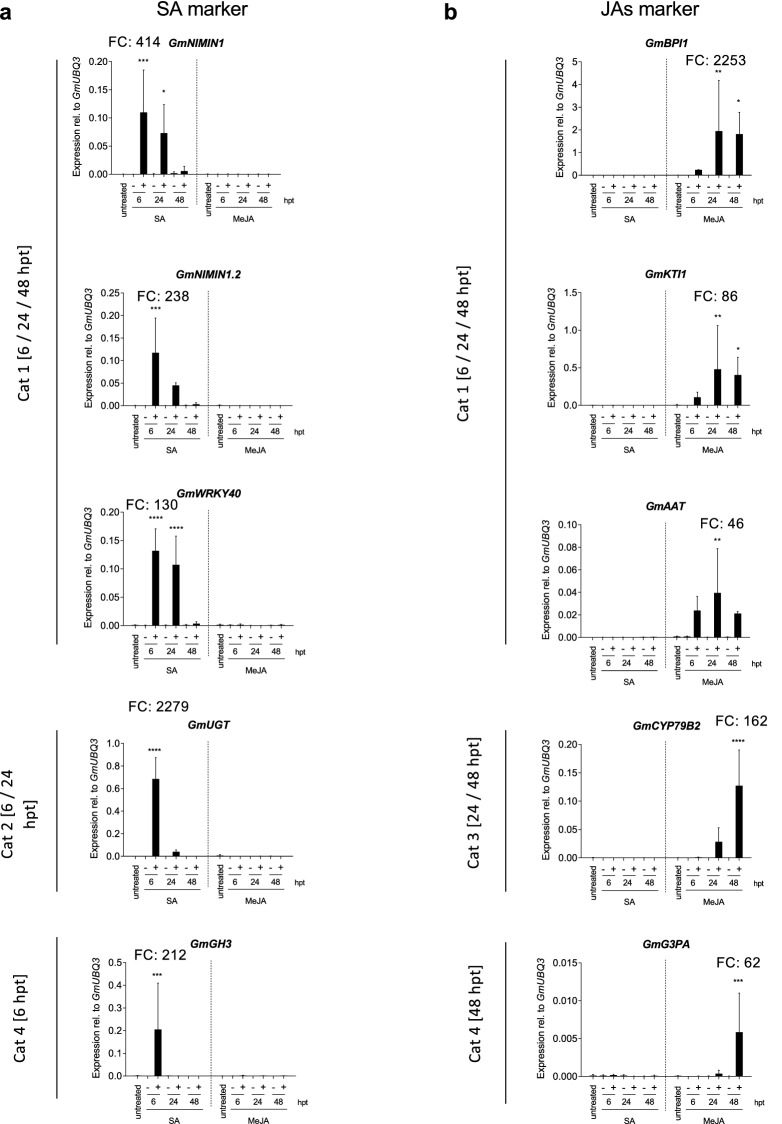
Verification of SA and JAs-induced marker gene expression in soybean leaves. Plants were either left untreated, subjected to control treatment (M) or treated with either SA or MeJA. The expression of selected marker genes was monitored in leaf samples at the indicated times post treatment (hpt) using qRT-PCR analysis. Asterisks indicate significant differences to the untreated control (Dunnett's multiple comparisons test; *p < 0.05, **p < 0.01, ***p < 0.001, ****p < 0.0001). FC indicates the maximum fold change in gene expression relative to the untreated control.

To evaluate the suitability of identified genes as specific molecular markers for SA or MeJA, we profiled the expression of each 5 selected candidate genes by qRT-PCR analysis. We focused on genes with strongest and/or longest-lasting activation upon treatment because we considered those genes as probably the most robust marker genes for the hormone in question. As seen in Figs. 2 and 3, detected expression kinetics of the selected genes were highly consistent between the RNA-seq and qRT-PCR analyses. Only SA, but neither control or MeJA treatment affected the expression of the selected SA-marker gene candidates *GmNIMIN1* (Glyma.10G010100)*, GmNIMIN1.2* (Glyma.02G009500), *GmWRKY40* (Glyma.17G222500) *GmUGT (Glyma.02G029900)* and *GmGH3* (*Glyma.17G165300*) (Fig. 3a). Expression of those genes was highly activated by SA with fold changes varying from ~ 200 to > 2000-fold (Fig. 3a) when compared to the untreated controls. In a similar manner, expression of the identified JAs-responsive genes *GmBPI1* (*Glyma.18G231700*)*, GmKTI1* (*Glyma.08G342000*),* GmAAT* (*Glyma.10G109500*)*, GmCYP79B2* (*Glyma.11G197300*) and *GmG3PA* (*Glyma.10G119900)* was triggered by MeJA, but not SA or mock treatment, with fold-changes in expression from ~ 60 to > 2000-fold over the untreated controls (Fig. 3b).

### Using identified marker genes to decipher soybean defense responses

**Figure 4:**
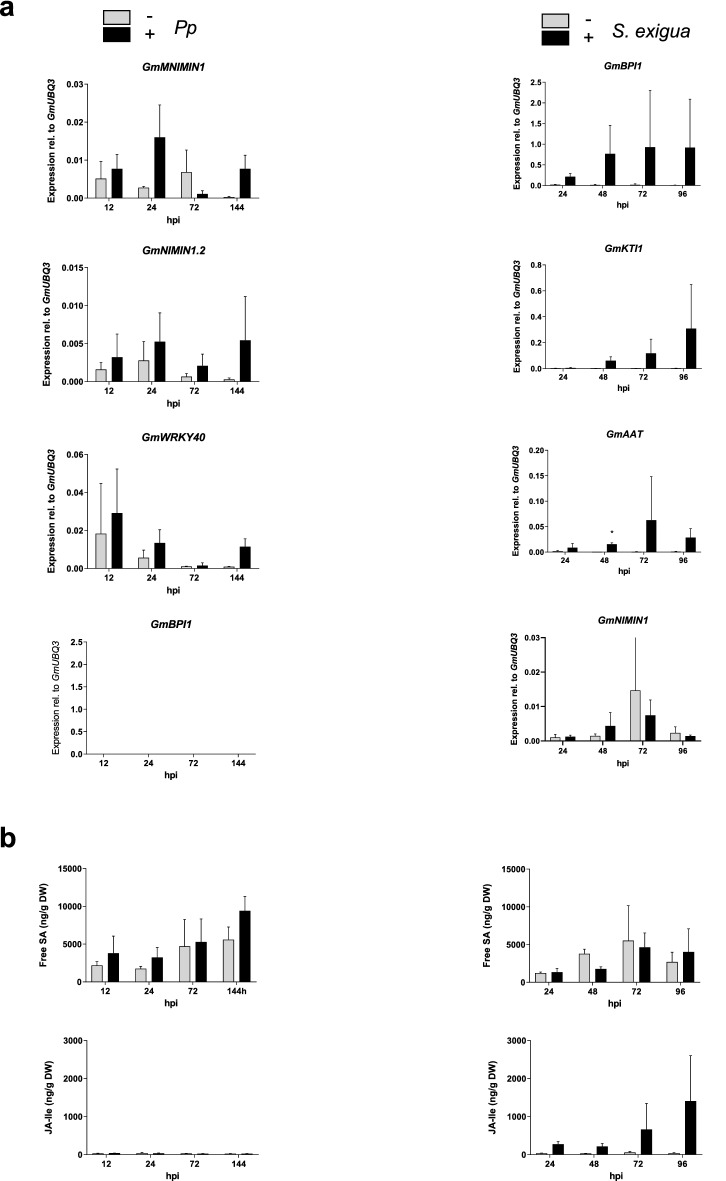
Correlation between the accumulation of free SA and JA-Ile and marker gene expression in soybean upon biotic stress. Expression of SA and JAs marker genes (**a**) and accumulation of SA and JA-Ile (**b**) in soybean leaves in a compatible interaction with *Pp* (left panel) or upon challenge with *S. exigua* larvae (right panel). To record hormonal and transcriptional responses upon challenge with *Pp,* first trifoliate leaves of intact soybean plants (W82, V2 stage) were either inoculated with a suspension of 1 mg ml^−1^ spores of *Pp* (isolate BR05) supplemented with 0.01% (v/v) Tween20 or treated with 0.01% (v/v) Tween20 only. To analyze the response of soybean plants to larvae feeding, first trifoliate leaves of W82 were subjected to 2nd–3rd instar larvae of *S. exigua* or left untreated (control). Hormone marker gene expression was quantified by qRT-PCR analysis and marker gene transcript abundance normalized to *GmUBQ3*. Identical samples were used for determining mRNA levels and quantification of the levels of free SA and JA-Ile by LC–MS. Asterisks indicate significant differences to the respective control (Sidak's multiple comparisons test; *p < 0.05).

To further verify the suitability of identified marker genes for analyzing recruitment of SA and/or JAs signaling in the soybean, we next compared the responsiveness of newly identified SA-marker genes and the level of SA in plants during a compatible interaction of soybean cultivar Williams 82 (W82) with the biotrophic fungal pathogen *Pp* (isolate BR05). To increase the chance of detecting possible transient accumulation of the hormone in question, we focused on marker genes in category 1, because genes in this category are induced for a longer period (Figs. 2 and 3). Both, *GmNIMIN1* and *GmWRKY40* showed a biphasic induction of expression in inoculated leaves of susceptible W82 plants with an apparent first expression maximum at 12–24 hpi and a second expression peak from 144 hpi whereas *GmNIMIN1.2* was expressed at higher levels in *Pp-*challenged than mock-inoculated leaves at all time points assayed (Fig. 4a). However, differences in expression of all three identified SA-marker genes among infected and mock-inoculated leaves were most distinct at 144 hpt. In contrast to the SA marker genes, expression of the top JAs-marker gene *GmBPI1* was not detected at any tested time point upon *Pp* inoculation (Fig. 4a).

To find out whether expression of presumed SA-marker genes would correlate with endogenous accumulation of SA, we quantified SA in leaves of *Pp*-infected and mock-inoculated soybean W82 plants. The highest content of free SA (~ 9.5 µg g^−1^ DW) was detected at 144 hpi after *Pp* inoculation (Fig. 4b). Free SA also accumulated early after inoculation (12–24 hpi). However, free SA levels at these time points remained below the accumulation at 144 hpi (Fig. 4b). At 72 hpi, however, free SA did not accumulate in response to *Pp* inoculation compared to mock controls. This observation is consistent with the expression of newly identified soybean SA-marker genes *GmNIMIN1* and *GmWRKY40* (Fig. 4a,b). In line with the absence of *GmBPI1* expression, accumulation of the most active JAs derivate JA-Ile was also absent at any time point tested after the *Pp* inoculation (Fig. 4a,b). Differential accumulation of free SA between mock and *Pp*-inoculated W82 leaves thus correlated with the abundance of mRNA transcript of adequate hormone marker genes (Fig. 4a,b).

Insect feeding often leads to the accumulation of JAs and to the activation of JAs-responsive defense genes in plants^41–43^. *Spodoptera exigua* is a generalist herbivore that feeds on soybean and many other plant species^44,45^. A detached leaf assay disclosed that feeding of *S. exigua* larvae caused activation of *GmAAT*, *GmBPI1* and *GmKTI1* gene expression in soybean (Fig. 4b). The expression of the three JAs-marker genes had an apparent maximum at 72–96 hpt (Fig. 4a). Of the three JAs-responsive genes, *GmBPI1* exhibited the strongest and most prolonged expression (Fig. 4a). To assess the specificity of identified marker genes for JA and SA we also quantified the level of transcript accumulation of the top SA-marker gene *GmNIMIN1* (Fig. 3)*.* In contrast to the JAs-marker genes, *GmNIMIN1* was not much responsive to feeding of soybean W82 leaflets with *S. exigua* larvae (Fig. 4a). In fact, expression of the gene was only slightly activated at 48 hpt upon feeding (Fig. 4a). Quantification of JA-Ile revealed a strong accumulation only after insect feeding with an apparent maximum of ~ 1.4 µg g^−1^ DW at 96 hpi (Fig. 4b). In contrast, free SA accumulated to higher levels in mock-treated leaves than upon insect-feeding (Fig. 4b). Thus, *GmNIMIN1, GmNIMIN1.2, GmWRKY40, GmBPI1, GmKTI1 and GmAAT* serve as reliable molecular markers to indirectly monitor hormonal changes upon insect feeding on soybean plants.

### Identification of genes for normalizing gene expression

For the precise determination of gene expression by qRT-PCR analysis and for adequate scientific conclusions reliable reference genes are required which are stably expressed across different experimental conditions. To identify such reference genes in soybean, we searched for genes that would display minimal variation in expression in all samples of our transcriptome dataset (untreated, mock-treated, SA/MeJA-treated). By doing so we identified 6 candidate genes with minimal changes in expression over all the samples (Table S3). The stability of expression of the genes was compared to the widely used reference gene *GmUBQ*^46–49^. Normfinder analysis^50^ disclosed that the 6 candidate reference genes were expressed with lower variably than *GmUBQ* in trifoliate leaves (Table S3). *Glyma.08G211200* was identified as the most stably expressed gene across all conditions tested in our analysis (Table S3). Normalizing the expression of the SA- and JA-marker genes *GmNIMIN1* and *GmBPI1* to the expression of *GmUBQ* and *Glyma.08G211200*, respectively, revealed a similar pattern of expression (Fig. S3). However, relative expression values were higher when normalized to the expression of *Glyma.08G211200* than *GmUBQ3* due to the overall lower expression level of *Glyma.08G211200* compared to *GmUBQ3* (Fig. S3). Our data, therefore, recommend *Glyma.08G211200* for use as a robust and lower expressed alternative to highly expressed *GmUBQ* for normalizing qRT-PCR-based evaluation of gene expression in soybean leaves, at least in the conditions used in this study.

## Discussion

Marker genes are powerful tools for characterizing the response of organisms to specific stimuli or in specific conditions. Although marker genes for the phytohormones SA and JAs are routinely used to monitor hormone pathway regulation in model plants, such as Arabidopsis, to our knowledge there is no empirical analysis that identified hormone-responsive genes as molecular markers in soybean. Selecting marker genes exclusively by their sequence homology to known hormone-responsive genes in other species can be misleading. This is particularly true for the paleopolyploid soybean, which has undergone at least two rounds of genome duplication and which possesses ~ 70% more protein-coding genes than Arabidopsis^51^. For example, in soybean there are multiple isoforms of the phenylalanine ammonia-lyase-encoding gene *PAL1.* Based on the available transcriptome data at SoyKB (http://soykb.org) genes of three PAL1 isoforms are not expressed. Shine and coworkers tested the expression of the five remaining soybean *PAL1* isoforms and they found that only two of them significantly responded to pathogen infection^52^. Furthermore, sequence homology to known SA, JA and ET marker genes in Arabidopsis and rice was used to identify presumably phytohormone-responsive genes in *Brachypodium distachyon*^53^. Only eight of 23 genes were activated by one of the hormones^53^. Consistently there was an overlap of only 26% and 42% of genes that responded to SA and JA, respectively, between *B. distachyon* and its closely related crop species rice^54^. These findings disclosed that sequence homology, especially in distantly related plant species, might be inappropriate for identifying reliable reporter genes.

Here, we experimentally identified marker genes that are specifically responsive to SA or JAs by simultaneously recording the transcriptome and hormone profile in leaves of soybean plants exposed to SA or MeJA. In addition, we mined our transcriptome data for genes that were stably expressed among all tested conditions to disclose robust reference genes with different expression intensity (Table S3, Fig. S3) for complementing the repertoire of frequently used, but often highly expressed, qRT-PCR reference genes in soybean, such as *GmUBQ*3^46–49^.

The rapid increase in free SA in the first trifoliate soybean leaves indicated responsiveness of the plant to the treatment (Fig. 1a). Elevated SA levels in leaves may either result from its rapid root-to-shoot transport or from de novo biosynthesis in distal leaves in response to SA as reported previously^55^ While basal levels of free and conjugated SA in soybean leaves (~ 0.5–1 µg g^−1^ FW; Fig. 1a) were similar to basal concentrations in tobacco (~ 0.2 µg g^−1^ FW), levels were higher in soybean leaves (up to ~ 100 µg g^−1^ FW; Fig. 1a) than in tobacco upon SA feeding (~ 5.7 µg g^−1^ FW)^56^. However, these differences correlate to the tenfold-higher concentration used in our study. Basal SA levels in soybean were also similar to those in Arabidopsis (0.25 µg g^−1^ FW to 1 µg g^−1^ FW) whereas SA content of potato and rice plants can be much higher (up to 10 µg g^−1^ FW and 37.19 µg g^−1^ FW, respectively)^57,58^. In tobacco and soybean, endogenously synthesized or exogenously supplied SA is quickly converted to presumably inactive SA 2‐*O*‐*β*‐d‐glucose (SAG) or salicylic acid glucose ester (SGE)^59–61^. The SA to SAG conversion likely minimizes possible negative impacts of high levels of free SA on plant fitness^62–64^. The glycosylation of free SA in soybean coincided with decreased levels of presumably bioactive, free SA and, thus, lesser genes were activated by SA at 24 and 48 hpt than at 6 hpt (Fig. 1a,c). In contrast, the accumulation of the bioactive JA derivative JA-Ile and global gene expression changes were highest at 24 and 48 h after exposure of trifoliate leaves to MeJA (Fig. 1). Measured levels of JA in soybean leaves reached from ~ 100 ng g^−1^ DW in untreated to ~ 40 µg g^−1^ DW in MeJA-exposed leaves while bioactive JA-Ile ranged between 0.1 and ~ 10 µg g^−1^ DW. Taking into account that dry weight was used for normalization in our study and assuming a water content of ~ 95%, JA concentrations in soybean leaves are in a similar range of what has been reported from intact and wounded Arabidopsis wild type plants (5–20 ng g^−1^ FW JA, respectively) or tobacco (40–300 ng g^−1^ FW JA, respectively)^65,66^. However, herbivore- (up to ~ 1 µg g^−1^ DW; Fig. 4b) or MeJA-induced levels of bioacticve JA-Ile (up to ~ 10 µg g^−1^ DW; Fig. 1b) in soybean markedly exceeded levels in wounded Arabidopsis (~ 2 ng g^−1^ FW JA-Ile) leaves^65^.

Our data disclose a quantitative correlation between increased in planta levels of bioactive hormone derivatives (JA-Ile and free SA) and downstream gene expression responses (Figs. 1c,d and 2). The high number of 334 genes activated exclusively at 6 hpt and the relatively low number of 19 genes activated at both 6 and 24 hpt, but the high overlap of 338 activated genes at 24 and 48 hpt (Fig. 1d) further indicate two distinct bursts of activated gene expression upon MeJA treatment in soybean. No such pattern was observed in response to SA (Fig. 1b). Two discrete waves of gene expression were also observed upon MeJA treatment of Arabidopsis cells^67^, although the expression peaked faster than observed here with soybean plants. Both, SA and MeJA enriched GO terms in bins according to their major functions in ‘SAR’ and ‘responses to wounding’, respectively (Fig. 1e,f)^41,68–72^. This indicates a conserved response of soybean and Arabidopsis to JAs and SA. Consistently, some soybean orthologs of SA and JAs-responsive Arabidopsis genes also specifically responded to respective hormone treatments of soybean plants [e.g. for SA: *PR1* (At2g14610, Glyma.15G062500, Glyma.15G062700), *PR2* (At3g57260, Glyma.03G132900); for JA: *VSP2* (At5g24770, Glyma.07G014600 and Glyma.08G200100)] (Table S4, Fig. S2)^22,23,27,28,73^ (Arabidopsis eFP Browser 2.0, http://bar.utoronto.ca/efp2/Arabidopsis/Arabidopsis_eFPBrowser2.html). However, other soybean orthologs of these genes [e.g. for SA: *PR1* (Glyma.15G062400), *PR2* (Glyma.19G134700 Glyma.19G134800 and Glyma.03G132700); for JA: *VSP2* (Glyma.08G200200)] and orthologs of other previously reported Arabidopsis hormone marker genes [e.g. for SA: *ALD1* (At2g13810, Glyma.08G180600) and *PAD4* (At3g52430, Glyma.06G156300, Glyma.04G209700); for JA: *VSP1* (At5g24780, Glyma.08G200000) and *PDF1.2* (At5g44420, Glyma.18G027700)] were either not, or not exclusively induced by JA or SA (Table S4). In soybean both *PAD4* genes were even repressed upon SA treatment which contrasts the reported SA-inducibility of *PAD4* in Arabidopsis^73^ (Table S4). Thus, despite some overlap between hormone-induced transcriptional responses in Arabidopsis and soybean, there are major differences in the hormone response in both plant species. Our data, therefore, discourage the utilization of marker genes that were exclusively selected by sequence homology to known marker genes in model plants without experimental validation. However, consistent with a widely conserved response of both plant species to SA, the Arabidopsis orthologs of the here identified SA-responsive soybean markers (*GmNIMIN1*, *GmNIMIN1.2, GmWRKY40, GmUGT and GmGH3*) encode proteins, or belong to protein families, that are closely associated with SA signaling in Arabidopsis. In fact, all of these genes or members of their gene families are reported to inhibit SA-responses either by directly depleting levels of free SA through conjugation with glucose or amino acids (members of the UGT and GH3 families, respectively)^59,74–76^ or, as is the case for the negative regulator of NPR1 functions NIMIN1 and also WRKY40, acting downstream of SA and eventually modulating the activation of SA-pathway associated genes^77–80^ to maintain immune homeostasis thus preventing an excessive defense response. Although it is tempting to speculate that the identified soybean SA-marker genes have similar biochemical functions as in Arabidopsis, functional characterization is needed to confirm their presumed function in the growth-to-defense trade-off in soybean.

We have some evidence for the SA-responsive marker gene *GmUGT* encoding an enzyme involved in SA homeostasis (Fig. S5). Transient overexpression of *GmUGT* in *Nicotiana benthamiana* prevented the accumulation of free SA in leaves of SA-treated plants whereas free SA accumulated to high levels in plants that did not express the transgene (Fig. S5). The biological importance of keeping SA responses in check is apparent by dwarfed mutants with deregulated SA levels, such as *cpr5*^81^. However, targeted mutation of the here identified soybean SA marker genes, or modulating their expression, may provide a promising strategy to engineer soybean plants with enhanced disease resistance especially to biotrophic pathogens via increased basal and/or pathogen-induced SA levels, or by amplifying downstream responses to SA accumulation. Although the boosted defense in such lines might be associated with enhanced fitness costs, reducing their disease susceptibility may outweigh putative growth penalties and provide increased yields especially in areas with high disease pressure.

Independent of their possible direct value for breeding the here identified marker genes provide novel tools for monitoring SA and JA-pathway activation in soybean to, e.g. characterize the impact of targeted or untargeted genetic modifications, treatments or conditions (e.g. biotic or abiotic stress) on the hormonal status in the crop. Indeed, there is a great overlap of genes with induced expression (> 1000 genes) when comparing the transcriptomes of SA-treated plants in our study to gene expression data of *GmMEKK1*-silenced soybean plants (Fig. S4) with constitutively elevated SA and defense gene expression levels^82^. Overlapping genes comprise the SA marker genes *GmNIMIN1*, *GmNINIM1.2*, *GmWRKY40* and *GmGH3*, supporting their responsiveness not only to externally applied but also endogenously accumulating levels of SA.

Consistent with the JAs responsiveness of proteinase inhibitor (PI) encoding *GmBPI1* and *GmKTI1* (Fig. 3), both genes were also activated in response to insect feeding or inoculation with necrotrophic pathogens^83–85^, two biotic stresses associated with activation of JAs signaling^3,4^. PIs are known for their potential to inhibit protein digestive enzymes in the insect gut^86^ pointing to a putative, but yet to be experimentally proven contribution of both candidate proteins to herbivore defense in soybean. The JAs marker gene *GmCYP79B2* was also reported to be higher expressed when soybean was exposed to bean pyralid larvae or high ozone in an ozone-resistant soybean cultivar^85,87^. Plant tolerance to high ozone levels has already been connected to JAs signaling^88^. In addition, JAs and ET-dependent induced systemic resistance^4,23^ is among the GO terms matching to *GmCYP79B2* which further supports its proposed applicability as a tool for monitoring JAs pathway activation. To our knowledge the JA-responsive genes *GmAAT* (*Glyma.10G109500*) and *GmG3PA* (*Glyma.10G119900*) have not been associated with JAs signaling so far.

To provide further experimental proof for the value of our identified marker genes we analyzed the expression of verified robust SA and JAs-responsive genes upon two biotic stresses that were expected to trigger distinct hormonal responses. Consistent with the paradigm of biotrophic pathogens predominantly activating SA responses, marker genes *GmNIMIN1*, *GmNIMIN1.2* and *GmWRK40* were induced upon soybean inoculation with the biotrophic fungus *Pp* whereas JA marker genes did not respond to this pathogen (Fig. 4a). Induction of SA marker gene expression but missing activation of JAs marker gene expression correlated with the presence of free SA and absence of JA-Ile in infected leaves, respectively (Fig. 4a,b). However, despite previous findings suggesting *Pp* to activate JA-responsive genes in Arabidopsis and soybean at early stages of infection^89–91^, we did not find any evidence for the rapid activation of JA signaling in soybean upon inoculation with *Pp*. Neither accumulation of JA-Ile nor JA-marker gene expression was detected at any time of sampling (Fig. 4). This might be different in interactions with Arabidopsis or of other *Pp* isolates with soybean cultivars other than the ones used here (*G. max* W82 and *Pp* isolate BR05, susceptible interaction) or when choosing different time points of sampling. Likewise, a more pronounced induction of SA and SA-responsive genes might be apparent e.g. in incompatible interactions with avirulent strains of the biotrophic fungus because SA accumulation is known to be associated with the hypersensitive response (HR)^65^. However, our data conform to the paradigm of biotrophic pathogens preferentially triggering SA- instead of JA-dependent defense responses in plants^92^. Conversely, challenge of soybean with *S. exigua* larvae caused a strong accumulation of JA-Ile and JA-marker gene transcripts whereas neither free SA levels nor downstream expression of SA markers were markedly altered upon insect feeding (Fig. 4). Hence, our study supports the application potential of the here identified hormone-marker genes to monitoring the dynamic accumulation of SA and JA, e.g. in particular stress conditions.

**Figure 5:**
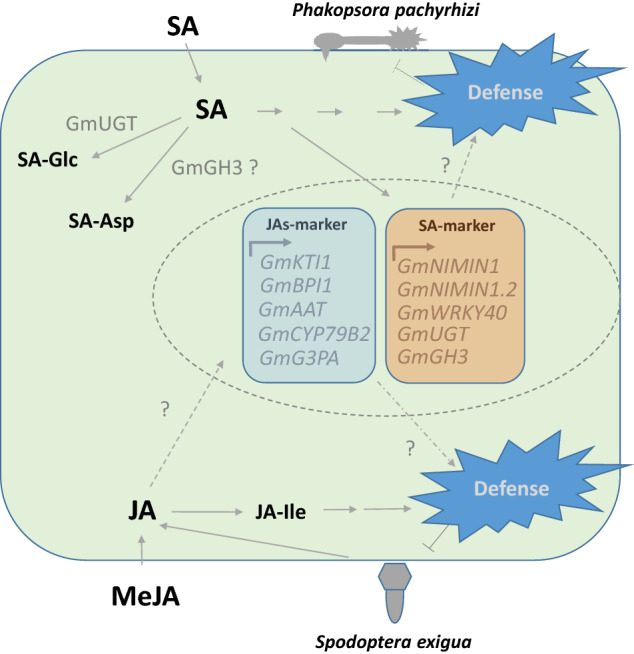
Molecular responses of soybean leaves to SA, MeJA, *P. pachyrhizi* attack, or *S. exigua* feeding. Treatment with SA or inoculation with *P. pachyrhizi* spores induces the consecutive accumulation of free and glycosylated SA and expression of SA-responsive genes with a suspected role in either the defense of biotrohic pathogens or SA homeostasis. JA and JA-Ile accumulate after MeJA exposure. They trigger the expression of JA-responsive genes with a presumed function in insect defense. *SA* salicylic acid, *SA-Asp* salicyloyl-aspartate, *SA-Glc* salicylic acid glucoside (SAG) or salicylic acid glucose ester (SGE), *JA* jasmonic acid, *MeJA* methyl jasmonate, *JA-Ile* jasmonic acid isoleucine.

Analyzing the responsiveness of genes simply selected by sequence homology to responding genes in other species can be misleading. Using whole transcriptome analyses we identified *GmNIMIN1*, *GmNIMIN1.2*, *GmWRK40*, GmUGT *and* GmGH3 as robust SA-responsive genes with hypothesized function in defense of SA-inducing biotrophic pathogens like *Phakopsora pachyrhizi* or SA homeostasis (Fig. 5). In addition, we disclosed *GmBPI1*, *GmKTI1*, *GmAAT*, *GmCYP79B2* and* GmG3PA* as the most reliable JAs-responsive transcripts with possible involvement of their encoded proteins in defense of JAs-inducing insect pests like *S. exigua*. Together, these sets of genes provide novel diagnostic tools to support research on disease and pest resistance in soybean and may help breeding and genetic engineering for improved soybean health.

## Methods

### Plant and fungal material

If not mentioned otherwise, *Pp* and soybean plants (genotype W82 [PI 518671]) were maintained and grown essentially as described^49^.

### Hormone treatments

Soybean plants were grown in sieved soil as described^49^ and treated with SA or MeJA in the V2 stage. For SA treatment by soil drench, pots with individual plants were placed in glass dishes (10 cm × 10 cm × 3 cm) to prevent cross contamination. The soil of each plant was drenched with 150 ml of a 1 mM SA solution in tap water (from a 200 mM SA stock in deionized water, pH 5.7). For control treatments deionized water (pH 5.7) without SA was added to tap water in the same way. After treatment, the plants were transferred to a growth chamber at the conditions described^49^. For each independent experiment, time point (6, 24, 48 h) and treatment (untreated, control, SA), three plants were used, of which the first trifoliate was harvested, trifoliates pooled and frozen in liquid nitrogen.

For MeJA treatment, two cotton balls of which each was soaked with 500 µl MeJA were placed in the middle of a closed transparent plastic container. Six soybean plants were placed around the cotton ball at equal distance, the container sealed and placed into a growth chamber at the conditions described above. Soybean plants that we used for control treatments were arranged the same way in a separate plastic container with two cotton balls but without MeJA. This container was placed in a separate growth chamber. For each independent experiment, time point (6, 24, 48 hpt) and treatment (untreated, control, MeJA), two plants were used, of which the first trifoliate was harvested, trifoliates pooled and frozen in liquid nitrogen. Control samples were always harvested before the hormone-treated samples.

### Extraction and quantification of phytohormones

Depending on the method used for quantifying hormone abundance (HPLC or UPLC-MS) we followed different extraction protocols.

SA was extracted and quantified by HPLC analysis according to the following protocol: ~ 100 mg homogenized frozen leaves were suspended in 700 µl of 90% (v/v) methanol. Upon addition of 700 µl of 100% (v/v) methanol the homogenate was spun in a centrifuge at RT for 15 min at 15,000×*g* and the supernatant transferred to a fresh tube. The supernatant was evaporated in a vacuum concentrator (Concentrator plus, Eppendorf) and the pellet suspended in 1 ml of 5% (v/v) trichloroacetic acid (on ice). Next, the samples were divided into two aliquots of which one was used for the quantification of free SA. The second aliquot was hydrolyzed for the quantification of total SA by adding 70 µl of concentrated HCl followed by incubation at 95 °C for 1 h. Both aliquots were then partitioned twice against 650 µl of ethylatetate:cyclohexane:2-propanole (50:49.5:0.5) by thorough mixing. After centrifugation for 5 min at 15,000×*g* the organic phase was transferred to a fresh tube and evaporated in the vacuum concentrator. Pellets were resuspended in 100 µl 100% (v/v) methanol. For SA quantification, 20 µl of the extracts were separated by HPLC on a reversed phase C18 column (KROMAPLUS 100-5-C18 5.0; Prontosil, BISCHOFF) for 15 min with a solvent flow of 1.5 ml min^−1^. Solvents and solvent gradient are shown in Table S5.

To quantify SA, JA, and JA-Ile by UPLC-MS, hormones were extracted by adding 2 ml of 10% (v/v) methanol to 50 mg of lyophilized, ground leaves. Samples were mixed and kept on ice for 45 min. Then, the samples were further homogenized on ice 3 times for each 20 s with a disperser (Ultra-Turrax, IKA Werk). After centrifugation for 45 min at 4000 rpm at 4 °C in a swing out rotor (3220 g) the supernatant was transferred to a fresh tube and the pH adjusted to ~ 2.5 with acetic acid. Samples were kept on ice during this step. They were then partitioned twice with 2 ml diethyl ether. After centrifugation for 3 min at 4000 rpm at 4 °C in a swingout rotor (4330 g). The organic phase was collected in a fresh tube and evaporated in a vacuum concentrator). Prior to measurement, extracted hormones were dissolved in 1 ml of MeOH/H_2_O with 0.01% HCOOH (10:90) containing a pool of internal standards of abscisic acid-d6 (ABA-d6), salicylic acid-d5 (SA-d5), indole acetic acid-d4 (IAA-d4), dehydro-jasmonic acid (dhJA), and jasmonic isoleucine-c6 (JA-Ile-c6) resulting in a final concentration of 100 ng ml^−1^. External calibration curves of each pure standard were prepared for the precise quantification ranging from 0.1 to 150 ppb. The quantification of phytohormones was performed in an Acquity ultraperformance liquid chromatography system (UPLC; Waters, Mildford, MA, USA) coupled to a triple quadrupole mass spectrometer (Xevo TQS, Waters, Manchester, UK). A UPLC Kinetex 2.6 µm EVO C18 100 Å, 2.1 × 50 mm (Phenomenex) column was used for the LC separation. Conditions and solvent gradients used in this chromatographic analysis were the same as described^93^.

### RNA extraction and qRT-PCR analysis

If used for RNA-seq, RNA was extracted from ground frozen leaves using the RNeasy Plant Mini Kit (Qiagen) according to the manufacturer’s instruction using the RLC buffer, stored at − 80 °C and shipped on dry ice for sequencing. For qRT-PCR analysis, RNA extraction and cDNA library preparation were carried out as described previously^49^. Primers for qRT-PCR were designed according to standard criteria^94^. For a list of used oligonucleotides, see Table S6.

### RNA-seq and marker gene identification

RNA quality control, library preparation, RNA-Seq and bioinformatic analysis were done by GATC-Biotech (Eurofins). A Genome sequencer Illumina HiSeq machine was used with a read length of 1 × 50 bp. Clean Reads were mapped to the *G. max* reference genome (genome assembly version Glyma.Wm82.a2 [Gmax2.0])^51^.

To identify SA and JAs-specific marker genes, we first selected genes that were significantly (p < 0.01) induced or repressed upon SA or MeJA treatment at 6, 24 or 48 hpt compared to the untreated control (t0). Furthermore, genes that were significantly induced or repressed at any time point in the controls compared to untreated control were discarded. We then assigned genes that fulfilled these criteria in accordance with their time point and duration of induced expression into different categories (1–4). Within these categories, genes were sorted by their average log2FC of the time points with significant induction compared to the untreated control. The top 50 genes in each category were then sorted by their average ΔFPKM value of the time points with significant induction compared to the untreated control to favor the selection of highly expressed genes. ∆FPKM values were calculated by subtracting average FPKM values from three independent experiments at an indicated time point after a treatment (6, 24, 48) or after control treatment (6_M, 24_M, 48_M) from the average FPKM-value of the untreated control. Shown top 15 genes of each category were sorted to their mean ΔFPKM-value across time points with significant induction.

### GO-enrichment analysis

For the GO enrichment analysis (available at: https://www.soybase.org/goslimgraphic_v2/dashboard.php, all genes that were significantly induced or repressed at any of the tested time points were used. Enriched GO terms were sorted by their corrected p-value from the lowest to the highest p-value.

### Exposure of soybean to biotic stress

Inoculation of soybean cultivar W82 with *Pp* isolate BR-05 was performed as described previously^49^. For the infestation of soybean (W82) with larvae of *S. exigua*, soybean plants were grown as described^49^. In V2 stage, the middle leaflet of the first trifoliate leaf was detached, the petiole covered with water-soaked paper tissue and placed in a petri dish. *S. exigua* larvae were kept on artificial diet (per liter: 28 g agar, 14 g polenta, 50 g yeast, 50 g wheat germ, 2 g sorbic acid, 1.6 g nipagin, 8 g ascorbic acid, 0.1 g streptomycin) for 7 days after hatching. Three larvae were placed on every leaflet and closed dishes were stacked and placed in plastic bag that was aerated once a day. Leaf tissue was harvested at the indicated time points after the infestation and frozen in N_2_.

### Cloning and transient expression

*GmUGT* was cloned from cDNA libraries of SA-treated soybean trifoliate leaves that were prepared as described above except using Oligo(dT) primers [TTTTTTTTTTTTTTTTTTTTVN]. The coding sequence of *GmUGT* (*Glyma.02G029900*) was amplified from cDNA using gene specific oligonuceotides (Table S6) and cloned into the pB7WG2D expression vector (https://gatewayvectors.vib.be/collection/pb7wg2d) by Gateway® cloning via pDONR207. The vector was transformed into the *Agrobacterium tumefaciens* strain AGL01 and used for transient expression in *N. benthamiana* as described^95^. GV2260 p19 helper strain was co-infiltrated to suppress gene silencing^96^. Control transformation was done by infiltrating the p19 helper strain only.

### Statements

All the methods were carried out in accordance with relevant guidelines and regulations. Furthermore, regulations and all experimental protocols were approved by a named institutional and/or licensing committee.

Seeds of soybean cultivar W82 were provided by the plant transformation facility at Iowa State University.

## Supplementary Information

Supplementary Information.
