# Supplementary material for: Disclosure of salicylic acid and jasmonic acid-responsive genes provides a molecular tool for deciphering stress responses in soybean
Source: Sci Rep. 2021 Oct 18;11:20600. doi: 10.1038/s41598-021-00209-6 (PMC8523552; doi:10.1038/s41598-021-00209-6)
Supplement: Supplementary file 1 — Supplementary Information. [file 41598_2021_209_MOESM1_ESM.pdf]

Treatment	Group	Sample name	Independent experiment #
None	Group 1	X1_0<br>X2_0<br>X3_0	#1<br>#2<br>#3
Mock; 6 h	Group 2	X1_6_M<br>X2_6_M<br>X3_6_M	#1<br>#2<br>#3
SA or MeJA treatment; 6 h	Group 3	X1_6<br>X2_6<br>X3_6	#1<br>#2<br>#3
Mock; 24 h	Group 4	X1_24_M<br>X2_24_M<br>X3_24_M	#1<br>#2<br>#3
SA or MeJA treatment; 24 h	Group 5	X1_24<br>X2_24<br>X3_24	#1<br>#2<br>#3
Mock; 48 h	Group 6	X1_48_M<br>X2_48_M<br>X3_48_M	#1<br>#2<br>#3
SA or MeJA treatment; 48 h	Group 7	X1_48<br>X2_48<br>X3_48	#1<br>#2<br>#3

X=A: SA treatment

X=B: MeJA treatment

Table S1. Assignment of labels to samples.

A

Sample	Total Reads	Discarded Reads	Clean Reads
A1_0	22,469,026	463,511 (2.1 %)	22,005,515 (97.9 %)
A1_24	47,612,366	994,399 (2.1 %)	46,617,967 (97.9 %)
A1_24_M	41,893,831	1,011,143 (2.4 %)	40,882,688 (97.6 %)
A1_48	27,622,395	262,047 (0.9 %)	27,360,348 (99.1 %)
A1_48_M	70,828,992	392,692 (0.6 %)	70,436,300 (99.4 %)
A1_6	41,145,982	1,011,595 (2.5 %)	40,134,387 (97.5 %)
A1_6_M	42,185,361	996,234 (2.4 %)	41,189,127 (97.6 %)
A2_0	23,917,715	520,786 (2.2 %)	23,396,929 (97.8 %)
A2_24	25,941,561	713,991 (2.8 %)	25,227,570 (97.2 %)
A2_24_M	24,018,786	510,167 (2.1 %)	23,508,619 (97.9 %)
A2_48	30,689,395	1,076,906 (3.5 %)	29,612,489 (96.5 %)
A2_48_M	28,817,415	609,234 (2.1 %)	28,208,181 (97.9 %)
A2_6	23,114,765	495,593 (2.1 %)	22,619,172 (97.9 %)
A2_6_M	20,167,713	478,534 (2.4 %)	19,689,179 (97.6 %)
A3_0	34,921,529	561,177 (1.6 %)	34,360,352 (98.4 %)
A3_24	45,766,784	734,775 (1.6 %)	45,032,009 (98.4 %)
A3_24_M	35,672,622	635,503 (1.8 %)	35,037,119 (98.2 %)
A3_48	37,508,352	607,421 (1.6 %)	36,900,931 (98.4 %)
A3_48_M	38,456,739	726,591 (1.9 %)	37,730,148 (98.1 %)
A3_6	42,731,513	770,416 (1.8 %)	41,961,097 (98.2 %)
A3_6_M	40,223,323	669,750 (1.7 %)	39,553,573 (98.3 %)

Sample	QC Passed Reads	Mapped Reads	% Mapped
A1_0	22,005,515	21,154,187	96.13
A1_24	46,617,967	44,846,589	96.20
A1_24_M	40,882,688	39,278,180	96.08
A1_48	27,360,348	26,219,682	95.83
A1_48_M	70,436,300	68,606,127	97.40
A1_6	40,134,387	38,437,383	95.77
A1_6_M	41,189,127	39,556,840	96.04
A2_0	23,396,929	22,785,283	97.39
A2_24	25,227,570	24,430,406	96.84
A2_24_M	23,508,619	22,822,303	97.08
A2_48	29,612,489	28,821,608	97.33
A2_48_M	28,208,181	27,420,568	97.21
A2_6	22,619,172	21,894,785	96.80
A2_6_M	19,689,179	19,193,322	97.48
A3_0	34,360,352	33,331,808	97.01
A3_24	45,032,009	43,707,399	97.06
A3_24_M	35,037,119	34,004,998	97.05
A3_48	36,900,931	35,772,529	96.94
A3_48_M	37,730,148	36,679,642	97.22
A3_6	41,961,097	40,652,340	96.88
A3_6_M	39,553,573	38,334,088	96.92

B

Sample	Total Reads	Discarded Reads	Clean Reads
B1_0	24,073,641	764,594 (3.2 %)	23,309,047 (96.8 %)
B1_24	27,296,062	516,053 (1.9 %)	26,780,009 (98.1 %)
B1_24_M	27,123,650	715,608 (2.6 %)	26,408,042 (97.4 %)
B1_48	22,805,116	860,894 (3.8 %)	21,944,222 (96.2 %)
B1_48_M	29,547,752	636,744 (2.2 %)	28,911,008 (97.8 %)
B1_6	30,536,841	620,546 (2.0 %)	29,916,295 (98.0 %)
B1_6_M	28,592,703	710,078 (2.5 %)	27,882,625 (97.5 %)
B2_0	24,155,560	563,715 (2.3 %)	23,591,845 (97.7 %)
B2_24_2	74,816,932	1,179,316 (1.6 %)	73,637,616 (98.4 %)
B2_24_M	33,867,979	630,814 (1.9 %)	33,237,165 (98.1 %)
B2_48	32,728,994	623,925 (1.9 %)	32,105,069 (98.1 %)
B2_48_M	28,202,504	735,948 (2.6 %)	27,466,556 (97.4 %)
B2_6	31,565,217	653,440 (2.1 %)	30,911,777 (97.9 %)
B2_6_M	28,897,083	606,990 (2.1 %)	28,290,093 (97.9 %)
B3_0	47,003,854	610,616 (1.3 %)	46,393,238 (98.7 %)
B3_24	31,651,033	619,112 (2.0 %)	31,031,921 (98.0 %)
B3_24_M	29,762,478	583,473 (2.0 %)	29,179,005 (98.0 %)
B3_48	28,212,937	721,551 (2.6 %)	27,491,386 (97.4 %)
B3_48_M	29,190,248	1,005,403 (3.4 %)	28,184,845 (96.6 %)
B3_6	28,512,232	730,266 (2.6 %)	27,781,966 (97.4 %)
B3_6_M	27,755,305	644,896 (2.3 %)	27,110,409 (97.7 %)

Sample	QC Passed Reads	Mapped Reads	% Mapped
B1_0	23,309,047	22,648,266	97.2
B1_24	26,780,009	25,964,078	97.0
B1_24_M	26,408,042	25,653,582	97.1
B1_48	21,944,222	21,279,167	97.0
B1_48_M	28,911,008	28,096,311	97.2
B1_6	29,916,295	28,966,718	96.8
B1_6_M	27,882,625	27,044,666	97.0
B2_0	23,591,845	22,850,691	96.9
B2_24_2	73,637,616	71,428,935	97.0
B2_24_M	33,237,165	32,334,617	97.3
B2_48	32,105,069	31,099,912	96.9
B2_48_M	27,466,556	26,732,343	97.3
B2_6	30,911,777	29,868,755	96.6
B2_6_M	28,290,093	27,416,575	96.9
B3_0	46,393,238	45,222,929	97.5
B3_24	31,031,921	30,005,629	96.7
B3_24_M	29,179,005	28,323,607	97.1
B3_48	27,491,386	26,556,365	96.6
B3_48_M	28,184,845	27,489,029	97.5
B3_6	27,781,966	26,821,587	96.5
B3_6_M	27,110,409	26,288,514	97.0

Table S2. Read statistics from RNA-Seq. Samples were derived from (A) SA treated, (B) MeJA treated or corresponding control or untreated plants.

Table S3

Gene name	Stability value
UBQ	0,027
Glyma.11G079600	0,026
Glyma.04G173300	0,012
Glyma.08G211200	0,009
Glyma.12G076100	0,009
Glyma.19G125600	0,011
Glyma.02G248700	0,013
**Best gene**	Glyma.08G211200
**Stability value**	0,009

Table S3. Stability values of *GmUBQ* and candidate *Glycine max* reference genes across all tested conditions as determined by NormFinder. The analysis is based on qRT-PCR data from three independent experiments.

Marker type	Gene name	Arabidopsis identifier	Soybean ortholog*	SA 6_M	SA 6	SA 24_M	SA 24	SA 48_M	SA 48	MeJA 6_M	MeJA 6	MeJA 24_M	MeJA 24	MeJA 48_M	MeJA 48
SA-responsive	PR1	AT2G14610	Glyma.15G062700, Glyma.15G062500	23,4	1061,9	-10,7	559,6	8,5	193,7						
			Glyma.15G062400	0,9	3,3	0,3	60,8	9,7	53,0	88,7	124,7	27,1	6,6	2,0	50,4
	PR2	AT3G57260	Glyma.19G134700	-0,2	-0,3	0,0	2,1		1,5	2,9	4,3	1,3	-0,5	1,0	2,3
			Glyma.03G132900	5,4	19,6	1,0	39,7	5,7	21,6	4,5	10,1	-3,3	-3,3	-1,6	-0,5
			Glyma.19G134800	0,9	0,7	-0,7	-0,2	-0,3	-0,1	2,3	3,9	-0,4	-0,5	-0,2	-0,4
			Glyma.03G132700	1,8	2,4	-26,0	-28,4	-28,6	-29,8	564,6	325,1	3,8	-4,5	-1,2	-13,0
	PAD4	AT3G52430	Glyma.06G156300	19,3	-13,9	6,4	-16,4	11,9	23,4	8,9	-7,1	-3,0	-15,4	-6,1	-10,8
			Glyma.04G209700	19,3	-13,9	6,4	-16,4	11,9	23,4	8,9	-7,1	-3,0	-15,4	-6,1	-10,8
	ALD1	AT2G13810	Glyma.08G180600	-0,1	1,0		2,7	1,0	1,4	1,4	2,8	1,1	0,2	3,1	7,0
JA-responsive	PDF1.2	AT5G44420	Glyma.18G027700												
	VSP1	AT5G24780	Glyma.08G200000	-1,6	-1,6	-1,7	-1,4	-1,5	-1,5						
	VSP2	AT5G24770	Glyma.08G200200	85,4	2,6	53,9	60,2	87,8	333,0						
			Glyma.07G014600	-10,4	-11,9	-12,7	-12,5	-10,5	-7,7	-5,9	27,9	-6,0	14,1	-5,9	-0,5
			Glyma.08G200100 (VSPB)	-8,7	-19,1	-5,1	-16,8	-9,1	1,7	-18,4	138,7	-27,6	820,8	-29,5	1253,0

ΔFPKM Color Code:

Color	Value
Yellow	10
Black	2
Blue	-2

Table S4. Expression pattern of soybean orthologs of hormone-responsive Arabidopsis marker genes upon treatment with SA or MeJA. Best-Hits-and-Inparalogs (BHIF)* were inferred from Blast hits against the PLAZA protein database (https://bioinformatics.psb.ugent.be/plaza/). Leaf transcriptomes were recorded by RNA-seq in SA, MeJA, control (M), or untreated soybean leaves at the indicated time points. Numbers correspond to ΔFPKM values that were highlighted according to the displayed color code. ΔFPKM values were calculated by subtracting average FPKM-values from three independent experiments at an indicated time point after a treatment (6, 24, 48) or mock treatment (6_M, 24_M, 48_M) from the average FPKM-value of the untreated control.

	Solvent (%)			
**time (min)**	**A**	**B**	**A:**	H_2O + 5% acetic acid
pre-run	60	40	**B:**	Methanol + 5% acetic acid
5,5	53,5	46,5		
7	40	60	**Flow rate:**	1 ml/min
17	35	65		
20	10	90		
25	10	90		
26	60	40		
30	60	40		

Table S5. HPLC gradient for SA quantification.

Name	Gene	Forward	Reverse	Type
GmNIMIN1	Glyma.10G010100	ATGTTGAACACGGCATTCTC	GGTACGGTGTGACTTTCTTG	
GmUGT	Glyma.02G029900	CCAAGCAGAATGGCCTTTAC	AACAGGGAGCTGAAGGAAAC	SA marker genes for qRT-PCR
GmG3H	Glyma.17G165300	GGCGCAGTATATTCCAACTC	GTTTGGCATGATGGTGTAGG	
GmNIMIN1.2	Glyma.02G009500	CCTGACACCAGACCATGATAC	TTATGGTTGTGCGTGGTTG	
GmWRKY	Glyma.17G222500	CACACCTAAAGGCTCATCAC	GTTTGAGACCACCCTGAAAG	
GmBPI1	Glyma.18G231700	TGAAGAAGGTGGCATTGG	GGAGCACCTGAGTAATGAAG	
GmKTI1	Glyma.08G342000	CTGCCGATCCGTATGATAAG	GGGACACAAGGCAGTATAAC	
GmAAT	Glyma.10G109500	GAGTTGATACCAGCCAACAG	GAGACCAACAAGCAGTGTAG	JAs marker genes for qRT-PCR
GmCYP79B2	Glyma.11G197300	AGGTACTTTGGGAAGGGTAG	CTCGAGCAAATGGAAGATGG	
GmG3PA	Glyma.10G119900	CGATGCACATGGTCTACTAC	CGATGGGAGCAGATAAAGAG	
GmVSP-B	Glyma.08g200100	GCTATGGTGAGCGTTCTTC	CCTGAAGAGTGCGTTGAAC	
GmNREG1	Glyma.11G079600	TCCTGGCTGCTAACTACTTG	GGTCTTGCGAATTTCCTCTG	
GmNREG2	Glyma.04G173300	GTGTCTCGCATGTTTCATCC	GTGAGTATAGCAGCCACATC	
GmNREG3	Glyma.08G211200	GCACTAGGAATGGCTATTGC	ACAGGTCTGCCTCTAGAATC	
GmNREG4	Glyma.12G076100	GATTGACTGCCAGTGATTCC	GCCTCAAACCTGAAGAATCC	Reference genes for qRT-PCR
GmNREG5	Glyma.19G125600	GTTGGGCATGAAATGGATGG	AACAGCTCGCCTCACTATAC	
GmNREG6	Glyma.02G248700	TGGAGAACCCAGAGCTTTAC	TAGAATGGCCTCCGTTTGTC	
GmUBQ3	Glyma.20G141600	GTGTAATGTTGGATGTGTTCCC	ACACAATTGAGTTCAACACAAACCG	
GmUGT	Glyma.02G029900	GGGGACAAGTTTGTACAAAAAAGCAGGCTCAATGGCTGAGCAGAGGCAAA	GGGGACCACTTTGTACAAGAAAGCTGGGTATCATATCAGATTCATCAAATGATTCAC	Cloning

Table S6. List of oligonucleotides used in this study. Gene-specific sequences of cloning primers are underlined.

Figure S1

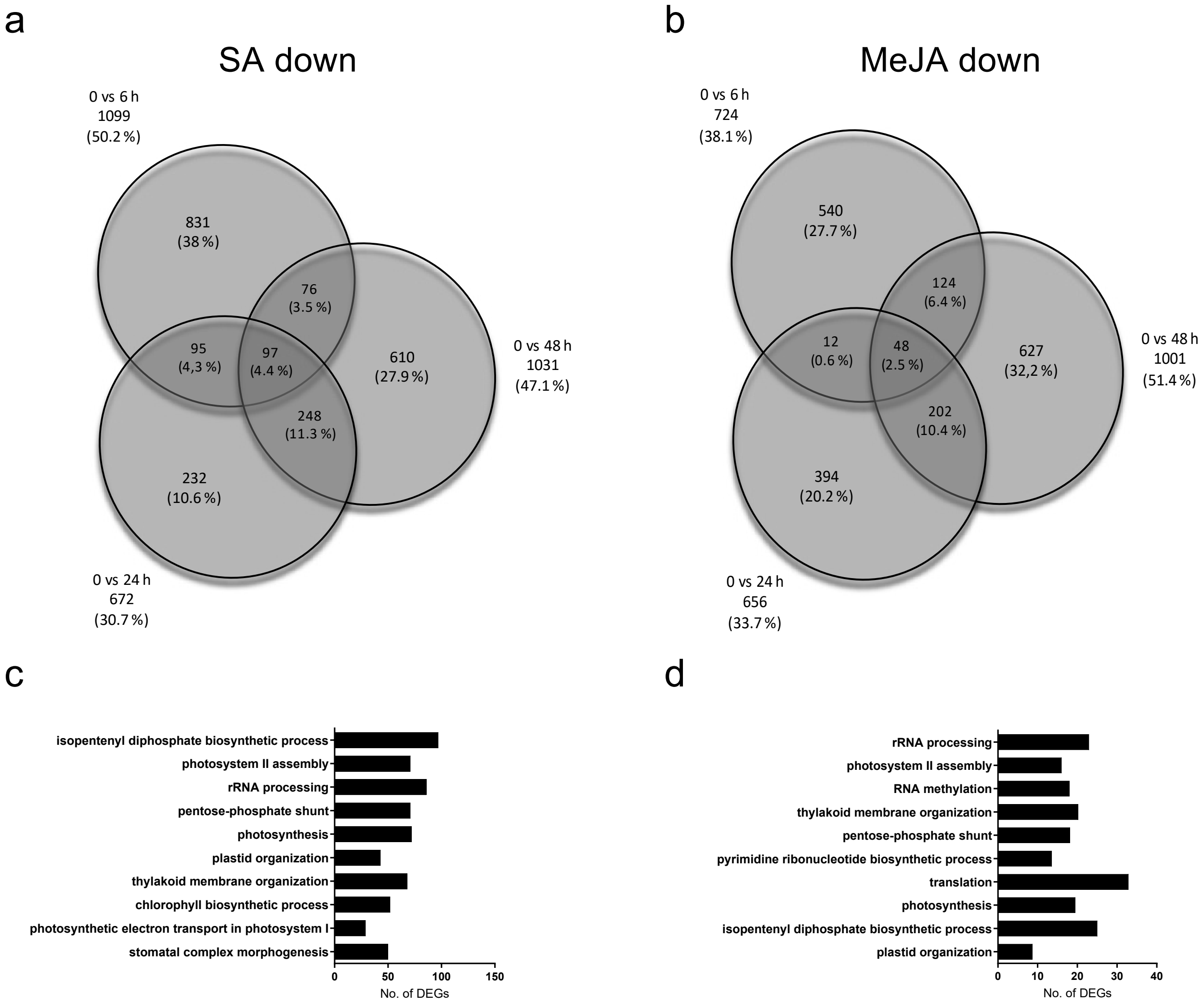

Figure S1. Venn diagrams displaying the number of genes with significantly ($p \leq 0.01$) repressed expression after (A) SA treatment and (B) MeJA exposure compared to untreated controls based on RNA-seq data. Only genes were included that were not differentially expressed among untreated and mock-treated samples. In addition to the absolute numbers, the relative amount of downregulated genes is shown in brackets. Corresponding levels of SA and JA are shown in Figure 1. (C-D) GO terms overrepresented among (C) SA-repressed (D) or MeJA-downregulated genes sorted according to their corrected p-value (Bonferroni correction) ($p<0.05$).

Figure S2

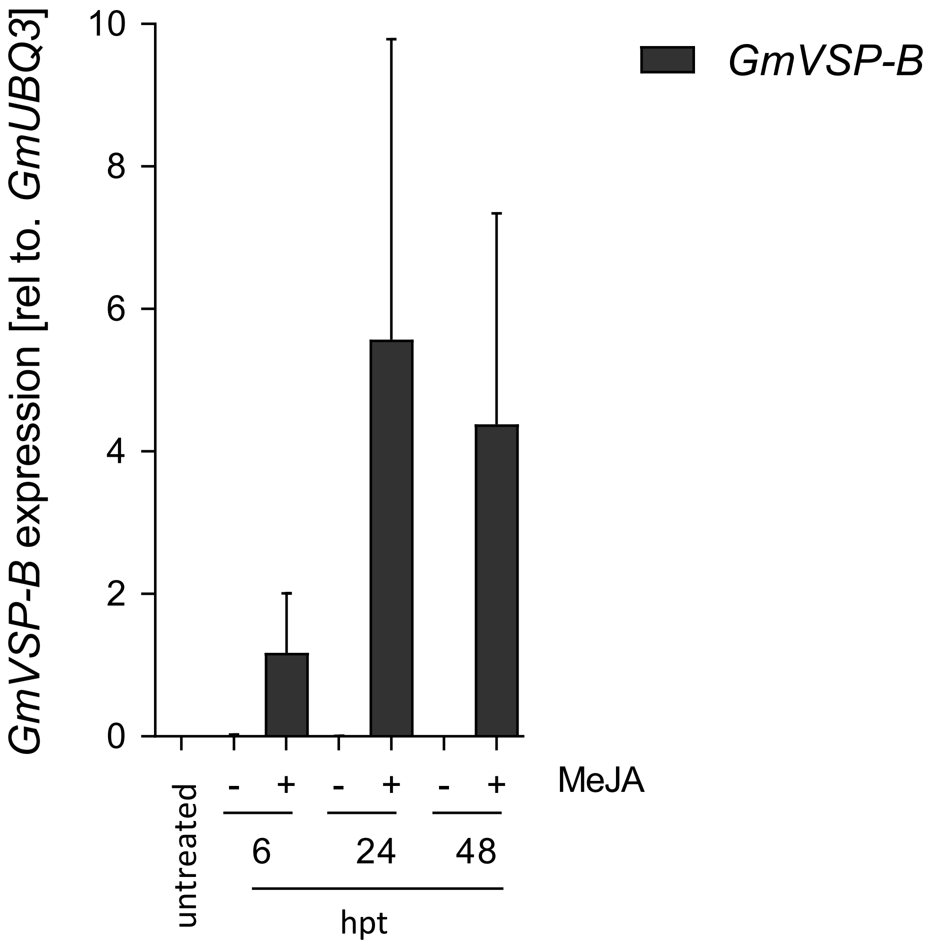

Figure S2. Expression of the designated JA-marker gene *GmVSP-B* (*Glyma.08G200100*) in leaves of MeJA-treated, control-treated (-) and untreated soybean plants. Expression of *GmVSP-B* was determined by qRT-PCR and normalized to the expression of *GmUBQ*.

Figure S3

a

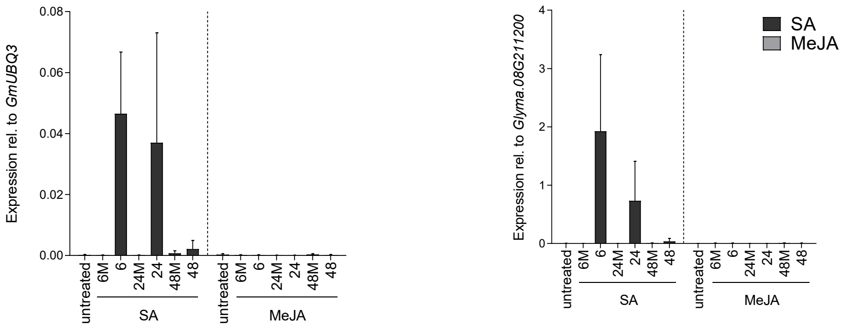

b

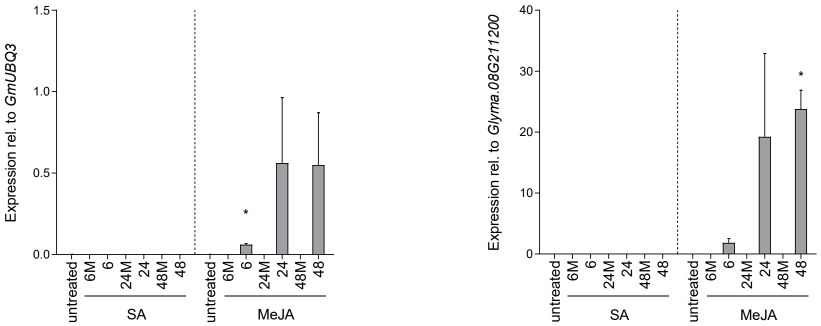

Figure S3. Expression pattern of SA- and JAs-responsive soybean marker genes when normalized to the expression of *GmUBQ* and *Glyma.08G211200*, respectively. Soybean plants were either left untreated, mock-treated (M) or treated with either SA or MeJA. Expression of *GmNIMIN1* (**A**, SA-marker gene) and *GmBPI1* (**B**, JAs-marker gene) monitored in leaf samples at the indicated times post treatment (hpt) by qRT-PCR using either *GmUBQ* or *Glyma.08G211200* as reference genes. Shown are mean values and SDEV from three indendent experiments. Asterisks indicate significant differences to the respective control (Sidak's multiple comparisons test; *= $p<0.05$)

Figure S4

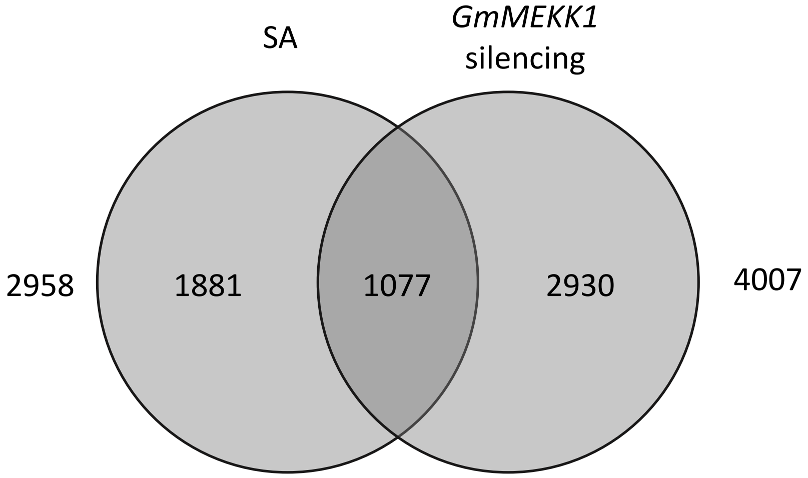

Figure S4. Overlap of significantly induced genes among SA-treated soybean (this study) and *GmMEKK1*-silenced soybean plants (Xu et al., 2018).

Figure S5

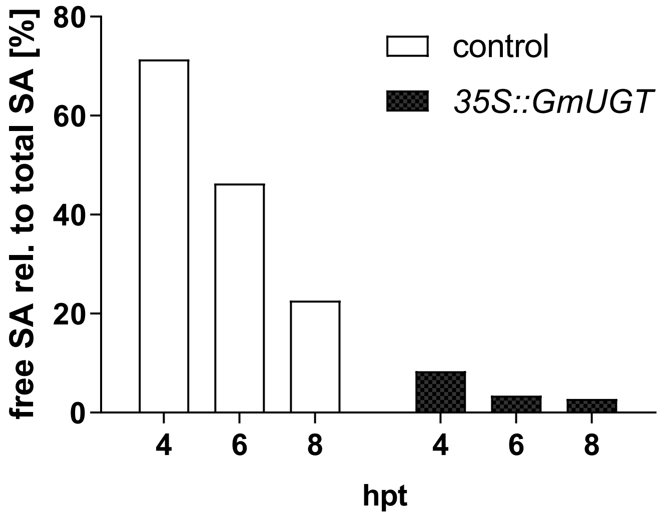

Figure S5. Transient overexpression of *GmUGT* in *Nicotiana benthamiana* reduces the ratio of free to total SA in leaves upon SA exposure. Leaves were transformed with a combination of *Agrobacterium tumefaciens* strains harboring a *GmUGT* and a p19 (silencing suppressor) overexpression construct, repectively, or infiltrated with the p19 strain only (control). Three days after transformation, 1mM SA was applied by soil drenching. Free and total SA were determined by HPLC at the indicated times after treatment.
